# Emphysematous pyelonephritis caused by *C. glabrata*

**DOI:** 10.1590/2175-8239-JBN-2020-0184

**Published:** 2021-03-24

**Authors:** Eduardo Augusto Schutz, Ana Paula Zabott, Rubia Bethania Biela Boaretto, Gisele Toyama, Carlos Floriano de Morais, Juliana Gerhardt Moroni, Carla Sakuma de Oliveira

**Affiliations:** 1 Universidade Estadual do Oeste do Paraná (Unioeste), Centro de Ciências Médicas e Farmacêuticas (CCMF), Hospital Universitário do Oeste do Paraná (HUOP), Cascavel, PR, Brasil.

**Keywords:** Urinary Tract Infections, *Candida* glabrata, Pyelonephritis, Nephrectomy, Diabetes *Mellitus*, Infecções Urinárias, *Candida* glabrata, Pielonefrite, Nefrectomia, Diabetes *Mellitus*

## Abstract

Emphysematous pyelonephritis (EPN) is a rare acute necrotizing infection of the kidney and surrounding tissues, with gas in the renal parenchyma, collecting system or perirenal tissue. The bacterial etiology predominates; mainly Gram-negative bacilli; *Candida* spp. and *C. albicans* are rarely described. We describe a case of EPN caused by *C. glabrata,* sensitive to fluconazole in a young, hypertensive woman with undiagnosed diabetes *mellitus* (DM), with renal dysfunction upon admission; her abdominal CT scan found a volumetric increase in the left kidney, signs of gas collections and perirenal blurring. Despite the antimicrobial therapy instituted, due to clinical refractoriness, a double J catheter and subsequent total nephrectomy were indicated, with good postoperative evolution. Her uroculture showed *C. glabrata* sensitive to fluconazole, and the pathology study showed tubular atrophy and intense interstitial inflammatory infiltrate. Despite the serious, potentially fatal condition, we could control the infection and the patient recovered fully. Poor DM management is an important triggering factor, and it is of great relevance to identify the EPN through imaging exams due to the peculiarities of its clinical and potentially surgical management

## Resumo

A pielonefrite enfisematosa (PNE) é uma infecção aguda rara necrotizante do rim e dos tecidos adjacentes, com presença de gás no parênquima renal, sistema coletor ou tecido perirrenal. Predomina a etiologia bacteriana, principalmente bacilos Gram-negativos; *Candida* spp. e na maioria das vezes *C. albicans* são raramente descritas. Descreve-se um caso de PNE causada por *C. glabrata* sensível a fluconazol em mulher jovem, hipertensa e com diabetes *mellitus* (DM) não diagnosticada, com disfunção renal à admissão; tomografia computadorizada de abdome constatou aumento volumétrico do rim esquerdo, sinais de coleções gasosas e borramento perirrenal. Apesar da terapia antimicrobiana instituída, devido à refratariedade clínica, foi indicado cateter duplo J e posterior nefrectomia total, com boa evolução pós-operatória. A urocultura evidenciou *C. glabrata* sensível a fluconazol, e o anatomopatológico demonstrou atrofia tubular e intenso infiltrado inflamatório intersticial. Apesar da condição grave, potencialmente fatal, houve controle do foco infeccioso e plena recuperação da paciente. O mau manejo do DM é um importante fator desencadeante, e é de grande relevância identificar a PNE por meio de exames de imagem devido às peculiaridades de seu manejo clínico e potencialmente cirúrgico.

## Introduction

Infectious diseases remain a major cause of morbidity and mortality worldwide, especially in developing countries. Emphysematous pyelonephritis (EPN) is a rare acute necrotizing infection of the renal parenchyma and adjacent tissues, resulting in the presence of gas in the renal parenchyma, collecting system or in the perirenal tissue^1^^,^ 2. Its etiology is mainly associated with Gram-negative bacilli, such as *E. coli*, *P. mirabilis* and *Klebsiella* spp; *Candida* spp. have rarely been reported as possible causes of EPN^2^^,^ 3.

*C. albicans* is the most frequent agent of candiduria, since it is part of the human oropharyngeal, gastrointestinal and genital tract microbiota^4^. Other species of *Candida* spp., such as *C. glabrata*, are not frequent in immunocompetent individuals; however, they can be found in patients with predisposing factors, such as diabetes *mellitus* (DM) or structural abnormalities of the kidneys and collecting system^5^. *C. glabrata* was not historically considered pathogenic, but there is a significant increase in reports of this agent in immunocompromised patients with urinary tract ( ITU) and systemic infections^6^.

With symptoms similar to acute pyelonephritis (fever, vomiting and low back pain), the evolution of EPN tends to be more serious, culminating in acute renal failure and septic complications, being potentially life threatening if not treated correctly and in a timely manner^3^. In this sense, the objective of this case report was to report the occurrence of EPN by *C. glabrata* in a diabetic patient, highlighting the clinical presentation and the treatment instituted, culminating in surgical removal of the affected kidney.

## Case Report

A 43-year-old female patient, previously hypertensive, was admitted to the emergency department of the Universitário do Oeste do Paraná (HUOP) Hospital with complaints of fever, diffuse abdominal pain, odynophagia and dyspnea for two days. On physical examination, she was hypertensive (140/100 mmHg), feverish (38 ° Celsius), with pain upon deep palpation throughout the abdomen, without signs of peritonitis, and edema of the lower limbs. Laboratory tests upon admission: leukocytosis 11,670/mm^3^, C-reactive protein (CRP) 23 mg/dL, creatinine 3.87 mg/dL, urea 170 mg/dL, blood glucose 231 mg/dL and glycated hemoglobin 14%; partial urine with 15 leukocytes/field and the presence of blastoconidium cells; chest radiography: obliteration of both costophrenic sinuses and bilateral interstitial diffuse infiltrate. With hypotheses of community-acquired pneumonia or pyelonephritis, we started her on clinical support with hydration and strict control of the newly diagnosed DM, and we prescribed moxifloxacin.

**Figure 1:**
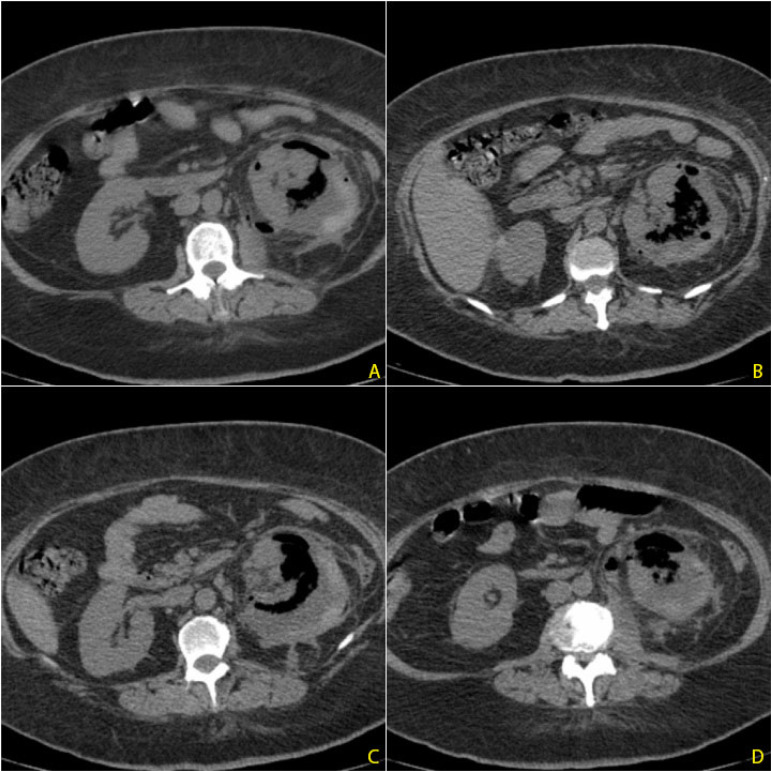
Computed tomography images of the abdomen without contrast. Panels A to D in cross section showing a volumetric increase of the left kidney, with signs of gas collections and perirenal blurring.

On the second day of hospitalization, there was a significant worsening of her odynophagia, and worsening of pain in the left flank with irradiation to the lower back, associated with positive wrist-percussion; upper gastrointestinal endoscopy, which demonstrated Kodsi II esophageal candidiasis. Her abdominal computed tomography (CT) scan revealed a volumetric enlargement of the left kidney with signs of gas collections and perirenal blurring (Figure 1), suggesting EPN. We then associated fluconazole to her treatment.

**Figure 2:**
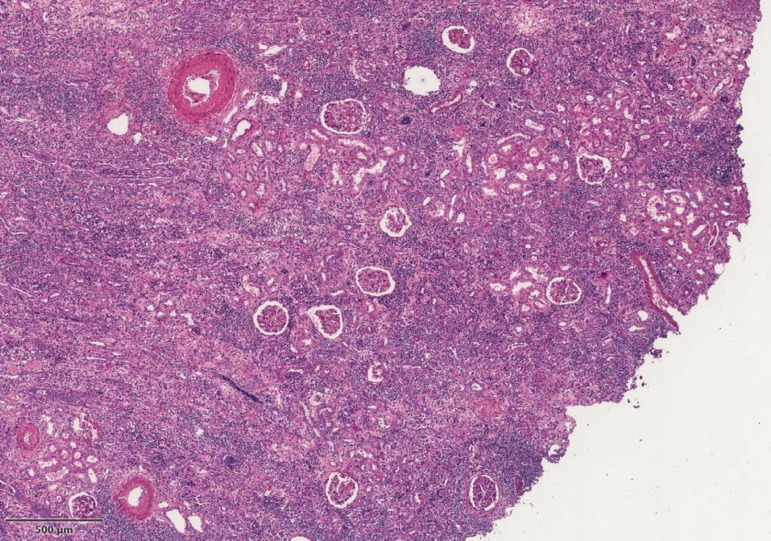
Hematoxylin and eosin staining in panoramic view of the cortical area showing tubular atrophy, extensive interstitial lymphoplasmocytic inflammatory infiltrate and intra-arterial thrombus, with preserved glomeruli.

On the fourth day, she remained feverish, with low back pain and poor general condition; her uroculture upon admission was negative for bacteria, but we decided to expand the antimicrobial spectrum using piperacillin with tazobactam. After two more days (D6), due to her persistent fever plus hydronephrosis, the urology team indicated urinary tract drainage with a double J catheter implantation. On the tenth day of treatment, despite a new negative urine culture for bacteria, she had a significant clinical deterioration, with decreased level of consciousness, severe abdominal pain and fever; we then indicated a surgical approach with total nephrectomy on the left. Her renal biopsy (Figure 2) revealed tubular atrophy with interstitial lymphoplasmocytic inflammatory infiltrate and a vessel with a thrombus inside.

Four days after the procedure (hospitalization D14), the patient evolved with significant clinical improvement, no fever, no abdominal pain and improved renal function (creatinine 0.92 mg/dL and urea 8 mg/dL), and she was discharged from the hospital, to finish with her treatment with fluconazole at home during the next 14 days. Upon her return to the outpatient clinic, she had fungi in her uroculture, with the growth of *Candida glabrata* sensitive to fluconazole.

## Discussion

EPN is a severe necrotizing kidney infection^1^^,^ 2, and it occurs mostly in patients with DM and in females; it commonly causes abdominal pain in the flanks, fever and pyuria^2^^,^ 3. In this study we present a case of EPN caused by *C. glabrata*, an unusual pathogen, with slow growth in culture. With the finding of concomitant esophageal candidiasis, we started her on an antifungal agent.

Factors related to its pathogenesis include: participation of gas-forming pathogens, tissues with a high concentration of glucose, impaired tissue perfusion and compromised immune system. Low oxygen tension in tissues with a high concentration of glucose induces anaerobic metabolism, with glucose acting as the main substrate for fermentation, releasing hydrogen gas (H2) and carbon dioxide (CO2) as a byproduct^7^^,^ 8. The patient was diabetic, without diagnosis and without previous treatment, presented this favorable microenvironment.

Diabetes *mellitus* (DM) increases susceptibility to infections due to the compromised immune response, and has multifactorial causes, such as reduced chemotaxis and opsonization, changes in cell adhesion to the epithelium, neutrophilic activity, production of cytokines by macrophages and compromised vascular supply^9^^,^ 10. These factors favor the transition from a commensal pathogen all the way to an overt infection^11^^,^ 12.

Candiduria is defined as 104-105 colony-forming units (CFU)/mL of yeasts detected in the urine, which may correspond only to a colonization of the urinary tract; UTI by *Candida* spp. is characterized by 105 CFU/mL in urine, associated with typical symptoms^4^^,^ 12. Its pathogenesis can be explained by colonization of the urinary tract and genital region or secondary to bloodstream infections^11^^,^ 12. Previous publications indicate that *Candida albicans* is the most frequently isolated species; however, an increase in the occurrence of non-albicans species of *Candida* has been reported, mainly due to the now common use of fluconazole^13^.

The antifungal of choice for UTI by *Candida* spp. is fluconazole^14^. Antifungal resistance is a current concern^9^, particularly in strains of *C. Glabrata* 15 ^,^ 16. For urinary tract infections caused by fluconazole-resistant *C. glabrata*, the drug of choice is Amphotericin B^17^. Still about this treatment, some controlled and randomized studies show that echinocandins can be considered options for the treatment of invasive candidiasis in non-neutropenic patients, suggesting an advantage in survival, with minimal adverse events^17^. In the case presented, fluconazole was used for the treatment of esophageal candidiasis, and in an empirical way for yeasts in the urine (considering *C. albicans* to be more frequent). When analyzing the culture with *C. glabrata* later in the outpatient clinic, there was a report of sensitivity to fluconazole, considering that the treatment was correct.

For the proper diagnosis of EPN, imaging tests are essential, especially the abdominal CT, which, in addition to allowing classification according to severity and prognosis, also detects possible stones and anatomical deformities in the urinary tract^3^^,^ 8. One of the main classifications, proposed by Huang et al.^8^, uses abdominal CT and divides EPN into four classes: 1) when there is gas confined to the collecting system, 2) gas confined to the renal parenchyma, 3A) presence of gas or abscess also in the perirenal space, 3B) presence of gas or abscess in the pararenal space, 4) bilateral EPN or a single kidney. We can therefore classify the present case as a class 3A EPN. 

Concerning treatment, patients should receive adequate support (hydration, sepsis protocols, DM control) and effective broad-spectrum antibiotic therapy. In patients with sepsis and two or more risk factors for poor prognosis (renal dysfunction, mental confusion, shock, and thrombocytopenia and polymicrobial infection), minimally invasive procedures for clearing the urinary tract, such as nephrostomy, ultrasound-guided aspiration or double J-catheter implantation should be considered. In cases of progressive clinical deterioration, nephrectomy for infection control should be considered, as long as clinical conditions permit^18^.

Sarvpreet et al. (2011) propose a treatment flowchart based on the abdominal CT classification in order to reduce mortality, since in many cases with indication of nephrectomy as the first option, mortality reached 50%. In the present case, conservative treatment was initially attempted with a broad-spectrum antibiotic and antifungal agent, but without clinical response. Even before surgery, the patient underwent double J catheter implantation (minimally invasive treatment), since she had a class 3A EPN with two risk factors, but definitive surgical treatment was necessary^3^.

## Conclusion

EPN is a serious, systemic infection, with a high potential for complication and death. As the case demonstrates, the poor management of DM is a trigger factor of great relevance. It is essential to identify the disease as soon as possible, in order to avoid the need for invasive treatments. A rare etiologic agent can contribute to failures in antimicrobial therapy; since she was diabetic, the findings of yeasts in the urine and esophageal candidiasis were determinant for correct preemptive antifungal therapy. In the case presented, due to the failure of clinical and minimally invasive treatments, probably due to her EPN classification (3A), definitive surgical treatment was necessary. The joint work of a multidisciplinary team (in this case: nephrology, infectious diseases, endocrinology, radiology, urology and pathology experts) was essential for the therapeutic success and the total recovery of the patient.
